# Bronchoscopic ICG-guided thoracoscopic segmentectomy for removal of an aspirated dental prosthesis in an elderly patient

**DOI:** 10.1093/jscr/rjaf866

**Published:** 2025-11-04

**Authors:** Eitetsu Koh, Yasuo Sekine, Fumihiro Ishibashi

**Affiliations:** Department of Thoracic Surgery, Tokyo Women's Medical University Yachiyo Medical Center, 477-96 Owada-Shinden, Yachiyo, Chiba 276-8524, Japan; Department of Thoracic Surgery, Tokyo Women's Medical University Yachiyo Medical Center, 477-96 Owada-Shinden, Yachiyo, Chiba 276-8524, Japan; Department of Thoracic Surgery, Tokyo Women's Medical University Yachiyo Medical Center, 477-96 Owada-Shinden, Yachiyo, Chiba 276-8524, Japan

**Keywords:** foreign body aspiration, dental prosthesis, indocyanine green, segmentectomy, thoracoscopic surgery

## Abstract

An 82-year-old male presented with persistent cough and recurrent pneumonia. Computed tomography revealed a high-density foreign body in the right lower lobe posterior basal segment (S10), later identified as a dental prosthesis. Bronchoscopic removal failed due to severe granulation and impaction. Because of low pulmonary function (FEV1: 920 ml), a lung-preserving procedure was selected. We performed thoracoscopic segmentectomy of S10, guided by bronchoscopic injection of indocyanine green (ICG) into B10a and B10b+c. Intraoperative near-infrared imaging clearly delineated the intersegmental plane. The foreign body was successfully removed, and the patient was discharged on postoperative day 7 without complications. This is the first reported case of bronchial ICG-guided anatomical segmentectomy for foreign body removal in an elderly patient. The technique allowed accurate resection with maximal preservation of lung function, demonstrating its clinical utility beyond oncologic indications.

## Introduction

Foreign body aspiration in the elderly is an uncommon but potentially serious clinical condition. While most cases are managed via bronchoscopy, certain impacted, or chronic foreign bodies may require surgical intervention. In such patients, particularly those with compromised pulmonary function, lung-preserving surgical techniques are preferred. Recent advances in fluorescence-guided surgery using indocyanine green (ICG) have enabled precise intersegmental demarcation during anatomical segmentectomy. Although commonly used in pulmonary oncology, its application for benign conditions, such as foreign body removal, is rarely reported.

## Case report

**Figure 1:**
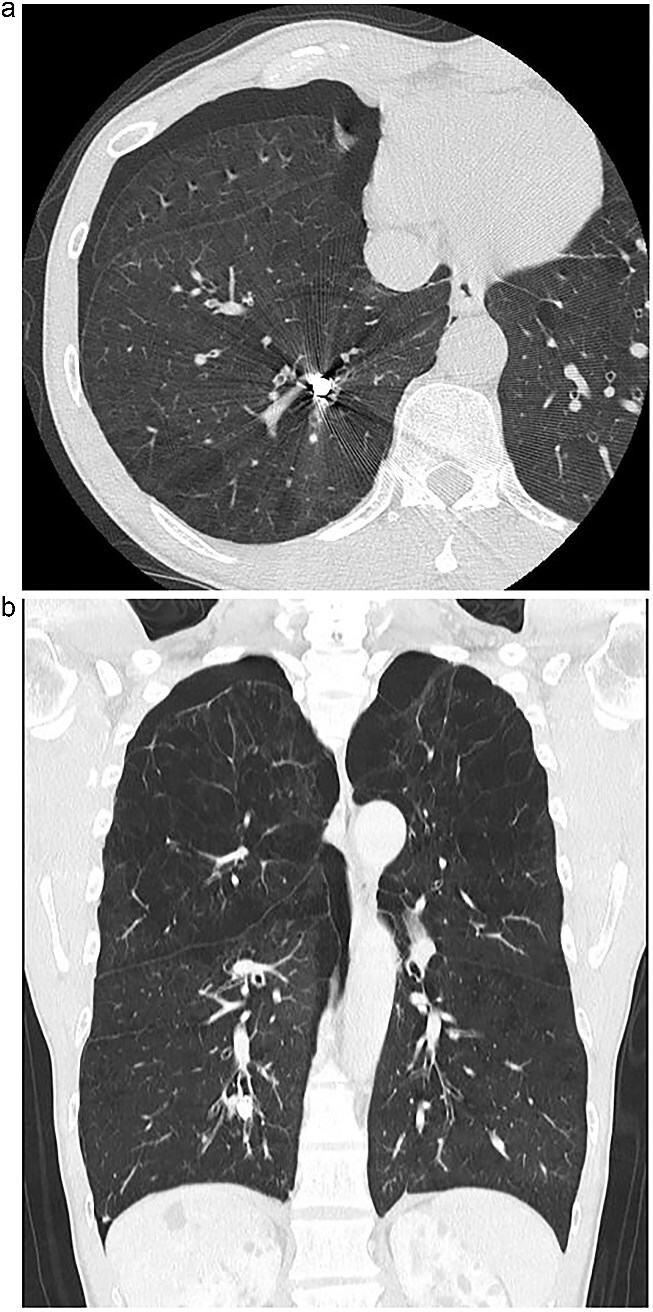
(a) Axial chest CT showing a high-density foreign body in the right S10 bronchus. (b) Coronal CT confirming the position of the dental prosthesis in the posterior basal segment.

**Figure 2:**
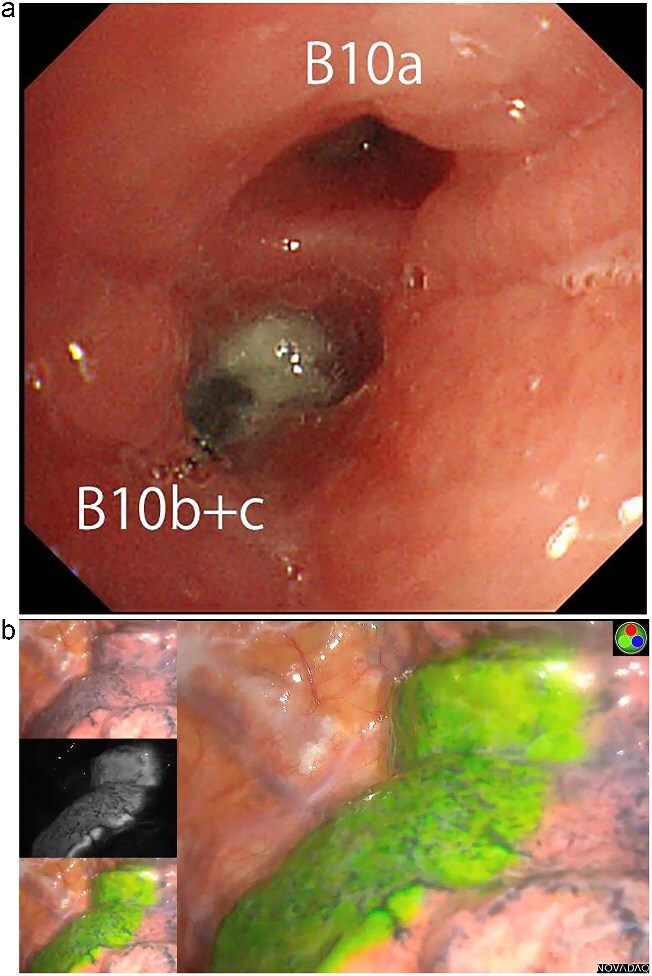
(a) Bronchoscopic image showing impacted dental prosthesis in the B10b+c bronchus. (b) Intraoperative near-infrared fluorescence image visualizing the intersegmental plane after ICG bronchial injection.

An 82-year-old man presented with chronic cough and a history of repeated right-sided pneumonia. Chest computed tomography (CT) showed a high-density object located in the posterior basal segment (S10) of the right lower lobe (Fig. 1a and b). Bronchoscopy revealed a dental prosthesis impacted in the B10b+c bronchus with surrounding granulation tissue (Fig. 2a). Removal via flexible bronchoscopy was unsuccessful due to firm impaction.

Pulmonary function tests showed reduced lung capacity (FEV1: 920 ml), making lobectomy undesirable. We therefore planned a thoracoscopic posterior basal segmentectomy (S10) to remove the prosthesis while preserving lung volume. To achieve accurate segmental resection, we adopted the bronchial ICG injection method, as previously described by Sekine *et al.* [1, 2]. ICG (25 mg diluted in 20 ml saline) was selectively injected into B10a and B10b+c via bronchoscopy. Near-infrared thoracoscopy was used to visualize the intersegmental plane clearly (Fig. 2b), and anatomical S10 segmentectomy was performed via thoracoscopy. The impacted dental prosthesis was successfully retrieved.

The patient’s postoperative course was uneventful. He was discharged on postoperative day 7. No complications occurred during the 2-year follow-up period, and no recurrence of pneumonia or respiratory symptoms was observed.

## Discussion

Foreign body aspiration in the elderly may go unnoticed for extended periods due to non-specific symptoms. While bronchoscopy is the first-line approach, chronic impaction and mucosal overgrowth can necessitate surgical retrieval. In this case, the foreign body could not be removed bronchoscopically due to granulation and impaction.

Given the patient’s limited pulmonary reserve, a minimally invasive and lung-preserving approach was critical. Segmentectomy allows for targeted resection with maximal preservation of lung parenchyma. The bronchial ICG injection technique offers a simple and reliable method for intersegmental plane identification. While intravenous ICG has been widely used, selective bronchial ICG instillation provides superior precision, particularly in patients with abnormal perfusion or prior inflammation.

This case demonstrates that bronchial ICG-guided segmentectomy is feasible and effective for non-oncologic indications, such as foreign body removal. It expands the clinical utility of this technique and supports its application in benign thoracic conditions, particularly in elderly or high-risk patients.

## Conclusion

Bronchial ICG-guided thoracoscopic segmentectomy enabled safe and effective removal of an aspirated dental prosthesis in a high-risk elderly patient. This technique may represent a valuable surgical option in similar cases, where bronchoscopy fails and parenchymal preservation is essential.

## Data Availability

All data supporting the findings of this case report are included within the article.
